# RpoS role in virulence and fitness in enteropathogenic *Escherichia coli*

**DOI:** 10.1371/journal.pone.0180381

**Published:** 2017-06-29

**Authors:** Gardênia Márcia Silva Campos Mata, Gerson Moura Ferreira, Beny Spira

**Affiliations:** Departamento de Microbiologia, Instituto de Ciências Biomédicas, Universidade de São Paulo, São Paulo-SP, Brazil; Centre National de la Recherche Scientifique, Aix-Marseille Université, FRANCE

## Abstract

Enteropathogenic *Escherichia coli* (EPEC) is a diarrheagenic pathogen that afflicts infants in developing countries. The most important virulence trait of EPEC is its ability to intimately adhere to cells in the small intestine, and to elicit diarrhea. The alternative sigma factor RpoS is involved in the virulence of several bacterial species. RpoS coordinates the general stress response and accumulates in cells under stress or in the stationary phase. RpoS levels differ across *E. coli* strains. High-RpoS strains are highly resistant to environmental stresses, but usually display low nutritional competence, while low-RpoS strains show the opposite phenotype. Here we investigated whether RpoS plays a role in the virulence and fitness of two different EPEC strains, E2348/69 and LRT9. A *rpoS* null mutation had a small positive effect on LRT9 adherence to epithelial cells, but the expression of the EPEC adhesins BfpA and intimin was not significantly affected by the mutation. E2348/69 adherence was not significantly affected by the *rpoS* mutation. The intrinsic level of RpoS was higher in LRT9 than in E2348/69 while the latter adhered more strongly and expressed higher levels of the adhesin BfpA than the former. Knockout of *rpoS* strongly impaired resistance to oxidative, osmotic and acid stress in both E2348/69 and LRT9. However, strain E2348/69 was significantly more sensitive to oxidative stress than LRT9. Finally, competition assays showed that the *rpoS* mutant of LRT9 displayed higher fitness under continuous culture than its isogenic wild-type strain, while E2348/69 outcompeted its *rpoS* mutant. In conclusion, RpoS plays mostly a positive role in EPEC biology and at least in the case of strain E2348/69 it is not constrained by the trade-off between vegetative growth and stress resistance.

## Introduction

The sigma factor RpoS is the master regulator of the general stress response in *E. coli* [1]. RpoS coordinates the transcription of genes associated with protection against environmental stresses, such as high osmolarity, oxygen free radicals, low temperature and others [1, 2]. Bacteria that lack RpoS are more sensitive to these stresses, thus though *rpoS* is not considered an essential gene, its presence strongly increases bacterial survival in stressful environments. *rpoS* is subject to diverse and multiple forms of control, been regulated at the transcriptional, translational and post-translational levels by many different inputs [3]. RpoS concentration increases under different situations, and is always associated with reduction in the growth rate. Accumulation of RpoS in the cytosol reduces the expression of growth-related genes due to the competition between RpoS and the vegetative sigma factor *σ*^70^ for a limited amount of RNA polymerase core units [4]. This characterizes a trade-off in which the bacterium sacrifices growth in favor of protection. This physiological adjustment deepens under prolonged starvation periods when mutations in *rpoS* or in genes that control *rpoS* expression are selected, resetting the SPANC (Self Preservation and Nutritional Competence) balance [5]. The *rpoS* gene is highly polymorphic and many different alleles are found in both natural isolates and laboratory strains of *E. coli* [6–9]. This strong variation is expected given the pivotal role of RpoS in the SPANC balance [5].

Enteropathogenic *E. coli* (EPEC) is a diarrheagenic lineage of *E. coli* that afflicts children in developing countries. Though not prevalent today as it was in the past, a significant number of cases of EPEC infection are still reported in Brazil and elsewhere [10–12]. EPEC strains are subdivided into typical and atypical strains [13]. Typical EPEC strains carry a large plasmid known as EAF, which harbors two operons (*bfp* and *perABC*) involved in the process of adherence to intestinal cells. The *bfp* operon is formed by 14 genes that are related to the biogenesis of the bundle-forming pilus (BFP), a type IV fimbriae [14]. *bfpA*, the first gene of the operon encodes the bundlin, the main subunit of the fimbriae BFP is needed for the first stage of infection and is responsible for the pattern of localized adherence (LA) to epithelial cells *in vitro* [15]. The first gene of the *per* operon, *perA*, encodes a positive regulator of *bfp* [16] and the product of *perC* induces the transcription of *ler* [17], which in turn is required for the expression of all operons present in the LEE, a pathogenicity island in the chromosome of EPEC and EHEC (Enterohaemorrhagic *E. coli*). The LEE genes are associated with the attaching and effacement lesion, which consists in the activation of the host cell signal transduction pathways and intimate attachment of the bacteria to the host epithelial cell [18].

RpoS affects the virulence of several bacterial species (for a recent review, see [19]). In pathogenic *E. coli*, the effect of RpoS on virulence is variable and sometimes conflicting. While some studies have shown that RpoS plays a positive role in the virulence of EHEC and in the expression of LEE [6, 20, 21], others have found that the opposite is true [6, 22, 23]. Another study has found that overexpression of *rpoS* in an EHEC *hfq* mutant did not have any effect on the expression of LEE [24]. In contrast, in *Citrobacter rodentium*, a bacterial model similar to EPEC that infects rodents, the transcriptional level of all LEE operons was enhanced by *rpoS* [6].

In the present study, the effect of *rpoS* on adherence, fitness and stress resistance of two EPEC strains was investigated. Both strains carry wild-type copies of *rpoS*, but express different levels of the RpoS protein. In both strains, RpoS did not play a considerable role in EPEC adherence to epithelial cells, but was absolutely required for bacterial survival under stressful conditions. The presence of *rpoS* had a small negative effect on LRT9 fitness, but did not impair the fitness of strain E2348/69.

## Materials and methods

### Strains, media and growth conditions

The strains used in this study are described in Table 1. The *rpoS*∷Tn*10* marker was introduced into E2348/69 and LRT9 strains by P1 transduction from strain MG1655 *rpoS*∷Tn*10* using phage P1 *vir* essentially as described [25]. LB medium/L-agar are as described [25]. T-salts medium (TGP) is a Tris-buffered minimal medium supplemented with 0.2% glucose and 1 mM KH_2_PO_4_ [26]. Dulbecco’s Modified Eagle’s Medium (DMEM) is a medium for epithelial cells (Cultilab-Brazil). HEp-2 cells were cultured in flasks containing DMEM enriched with 10% fetal calf serum (FCS), 50 U penicillin and 50 μg/ml streptomycin at 37℃. Antibiotics were omitted in assays whenever bacteria were added. For overnight growth, bacteria were usually cultivated in LB medium, for all other purposes they were grown in either TGP or DMEM. Growth rate was calculated according to the formula: $\mu =\frac{ln\frac{N}{N_{0}}}{t}$, where *N* and *N*_0_ respectively correspond to the initial and final *OD*_600_ at the exponential growth phase and *t* is the time-course of the growth curve.

**Table 1:** Bacterial strains and plasmids used in this study.

**Strains**	**Genotype**	**Source**
E2348/69	EPEC 0127:H6 Nal^R^	[27]
CFP1	E2348/69 *rpoS*∷Tn*10*	This study
LRT9	EPEC O111:abH2	[28]
GMF237	LRT9 *rpoS*∷Tn*10*	This study
LG01	LRT9 *lacZ*∷Tn5	Lab collection
MC4100	F- *ara*D139 (*argF-lac*)U169 *rpsL*150 *deoC*1 *relA1 thiA ptsF*25 *flbB*5301 *rbsR*	[29]
BS878	MC4100 *rpoS*∷Tn*10*	This study
BS1230	LRT9 *bfpA*∷SPA-Km	[30]
BS1332	GMF237 *bfpA*∷SPA-Km *rpoS*∷Tn*10*	This study
BS1298	LRT9 *eae*∷SPA-Km	[30]
BS1307	GMF237 *eae*∷SPA-Km *rpoS*∷Tn*10*	This study
**Plasmids**	**Relevant feature**	**Source**
pRK*lacZ* 290	Low copy vector carrying a promoterless *lacZ*	[31]
pGM30	*bfpA* promoter cloned upstream of *lacZ* in plasmid pRK*lacZ* 290-SpR	This study
pGM36	*tir-eae* promoter cloned upstream of *lacZ* in plasmid pRK*lacZ* 290-SpR	This study
pNP5	*rpoS*^+^ cloned in the low-copy plasmid vector pACT3	[32]

### Stress assays

Bacteria grown overnight in LB medium were challenged as follows. For the acid stress, 4 × 10^3^ cells from the overnight culture were suspended in 1 ml EG buffer (0.4% glucose; 73 mM K_2_HPO_4_; 17 mM NaNH_4_HPO_4_; 0.8 mM MgSO_4_; 10 mM citrate; 1.5 mM glutamate; pH 2). Aliquots were removed every 5 minutes up to 20 min and plated on L-agar. Oxidative stress was induced by treating a bacterial suspension in 0.9% NaCl containing 4 × 10^3^ cells with 6 mM H_2_O_2_ for 5, 10, 15 and 20 minutes and subsequently plated. 4 × 10^3^ bacteria were subjected to osmotic stress by incubating for 0, 2, 4 or 6 hours in a 2 M NaCl solution. All plates were incubated overnight at 37℃ followed by CFU counting. The results are shown in percentage of the number of CFU/ml, with the CFU at time 0 being 100%.

### Sequencing *rpoS*

The *rpoS* ORF of E2348/69 and LRT9 was each amplified by PCR using primers rpoS-429F (5’–GGAACAACAAGAAGTTAAGG)/rpoSb-E2348 (5’–TGATGAACACATAGGGTGCAA). For the sequencing reaction, besides rpoS-429F and rpoSb-E2348, primers rpos9363+ (5’-CATACGCAACCTGGTGGATT), rpoStr-EcoRI (5’-GTGATAACGAATTCGCCGAAGAGG) and rpoS1421 (5’- TCGAACAGCCATTTGACGATG) were also used. The PCR products were purified using the Concert Rapid PCR Purification System kit (Life Technologies, Bethesda, MD). Sequencing reactions were directly performed from purified PCR products using the same primers for both strands and Big Dye Terminator v3.1 (Life Technologies, Foster City, CA). Sequencing was carried on an automated sequencer (ABI Prism 3130XL DNA Analyzer, Applied Biosystems, Foster City), according to the manufacturer recommendations.

### Immunoblotting

Bacteria grown overnight in TGP containing 0.2 mM KH_2_PO_4_ (limited Pi concentration) were centrifuged, and a culture volume corresponding to an OD_600_ of 1.0 (approx. 10^9^ cells) was resuspended in 0.1 ml Application Buffer (0.5 M Tris/HCl, 2% SDS, 5% 2-mercaptoethanol, 10%, v/v, glycerol and 0.01% bromophenol blue) and boiled for 5 min. Ten μl samples were resolved by standard SDS-PAGE (12.5% acrylamide). Following electrophoresis, proteins were transferred to a nitrocellulose membrane using a Trans-blot Semi-Dry Transfer Cell (BioRad, USA), as recommended by the manufacturer. The membrane was subjected to blocking with 5% skimmed milk and exposed to anti-FLAG M2 (Sigma) monoclonal antibodies, anti-RpoS (Neoclone) monoclonal antibodies (1,000X dilution) or anti-RpoD (Santa Cruz) monoclonal antibodies (5,000X dilution), followed by exposure to anti-mouse IgG serum conjugated to peroxidase (Thermo Scientific) diluted 10,000-20,000. Membranes were developed using the Clarity Max detection kit (Bio-Rad) and read in the Bio-Rad Imaging System.

### Adherence assay competition

Approximately 10^5^ HEp-2 cells (ATCC^®^ CCL-23™) were added to each well of a 24-well tissue plate and grown for 48 h at 37℃ with 5% CO_2_. The medium was removed from the cell monolayer and replaced with 1 ml of fresh DMEM supplemented with 2% FCS and 1% mannose. At this point, 5 × 10^7^ bacteria of each strain (E2348/69, LRT9 or their respective *rpoS*∷Tn*10* mutants) previously grown overnight in LB were mixed in pairs and added to each well. After 3 h of incubation, the cell monolayer was washed six times with phosphate-buffered saline (PBS) to remove the non-adherent bacteria. The monolayer containing the adhered bacteria was treated with 1 ml 0.1% Triton X-100 in PBS for 5 minutes, bacteria were further diluted in PBS, plated onto L-agar, L-agar supplemented with tetracycline (that allows the growth of E2348/69 *rpoS*∷Tn*10* and LRT9 *rpoS*∷Tn*10*) or ampicillin (LRT9 is naturally resistant to Amp) and incubated at 37℃ for 24 h. On the next day, the number of colony forming units per ml (CFU/ml) for each competing strain was calculated.

The adherence assay shown in S1 Fig was performed as described above, except that the bacterial strains were not mixed.

### RNA extraction and northern blotting

Bacteria were grown in DMEM without agitation at 37°C up to an OD_600_ of ∼0.5 (exponential phase) or to the beginning of the stationary phase (OD_600_ ∼1.0). RNA was extracted essentially as described [28]. Twenty micrograms of total RNA were resolved by electrophoresis in an 1% agarose gel containing 7% formaldehyde and transferred to a nylon membrane by capillary force. A ^32^P-labeled *bfpA* DNA probe was synthesized by random primer labeling using ^32^P-dCTP. The DNA template was obtained by PCR amplification using the oligonucleotides bfp-A (5’-AATGGTGCTTGCGCTTGCTGC) and bfp-B (5’-GCCGCTTTATCCAACCTGGT). The membranes were hybridized with the labeled probes at 42℃ in hybridization solution (MRC- HS114F) for at least 16h, washed and exposed to X-ray films.

### Construction of *bfpA-lacZ* and *eae-lacZ* fusions

The LEE5 (*tir-eae-cesT*) promoter region was amplified by PCR using the genomic DNA of EPEC E2348/69 as a template and primers tir-P1 (AGTGGATCCCATTACACGTTTT) and tir-P2 (CCGTCTGTTTGTGAAGGTAGTG). The promoter region of bfpA was amplified using the E2348/69 EAF plasmid and primers bfp-P1 (GCACTGGTCATGGATACAGTT) and bfp-P2 (TCAGACGCAGACTGGTAGTAA). The PCR products we first cloned in pGEM T-Easy (Thermo), digested with EcoRI and subcloned in plasmid pRK*lacZ* 290. The orientation of the cloned fragments was determined by sequencing. A spectinomycin resistance cassette was excised from plasmid pJL74 [33] and ligated to the EcoRV site inside the tetracycline-resistance gene of the pRK*lacZ* 290 derivatives, originating plasmids pGM30 and pGM36.

### β-galactosidase assay

β-galactosidase assays were carried out in microplates essentially as described [34]. Briefly, culture aliquots grown in a 24-well microplate were collected and transferred to a 96-well plate containing 80 μl of a freshly prepared permeabilization solution (100 mM Na_2_HPO_4_, 20 mM KCl, 2 mM MgSO_4_, 0.6 mg/ml hexadecyltrimethylammonium bromide (CTAB), 0.4 mg/ml sodium deoxycholate, 2.7 mM tris(2-carboxyethyl)phosphine (TCEP) and carbenicillin). 25 μl of each sample were transferred to a new 96-well microplate. The assay was initiated by adding 175 μl of a freshly prepared substrate solution (60 mM Na_2_HPO_4_, 40 mM NaH_2_PO_4_, 4 mg/ml o-nitrophenyl-β-D-Galactoside (ONPG) and 1.35 mM TCEP) to the permeabilized cells. The plates were briefly centrifuged to minimize the formation of bubbles and the OD_550_ of the samples was determined. The reaction was monitored by reading the plates at A_420_ every 15 min at room temperature until a yellow color was developed. Each point corresponds to at least three independent cultures, and each culture was assayed twice. Miller units were calculated as described [25]: $1\;Miller\;Unit\;=\;1000\;\times \;\frac{\left(A_{420}-\left(1.75\times OD_{550}\right)\right)}{\left(t\times v\times OD_{600}\right)}$, where A_420_ stand for the absorbance of the sample at 420 nm, OD_550_ and OD_600_ record the turbidity of the sample at the specified wavelengths; *t* is the reaction time; and *v* is the volume of assayed culture.

### Competition assays

Competition assays between the strains E2348/69, LRT9 and MC4100 with their respective *rpoS* mutants were performed under continuous culture in TGP medium supplemented with 0.2% glucose and 30 μM KH_2_PO_4_ at 37℃ for 24 hours. The competition was started by mixing equal concentrations of each strain (at an OD_600_ = 0.01). The chemostat was set at a dilution rate of 1.0 h^-1^. Samples were taken at time zero and after 24 hours and plated on L-agar and L-agar supplemented with tetracycline. CFU counting of each strain was determined following overnight incubation at 37℃.

### Statistical analysis

The standard error of the mean was calculated according to the formula $SEM=\frac{SD}{\sqrt{n}}$, where SD is the standard deviation [35]. Data were evaluated for statistical significance using a two-tailed heteroscedastic Student’s t-test.

## Results

**Fig 1:**
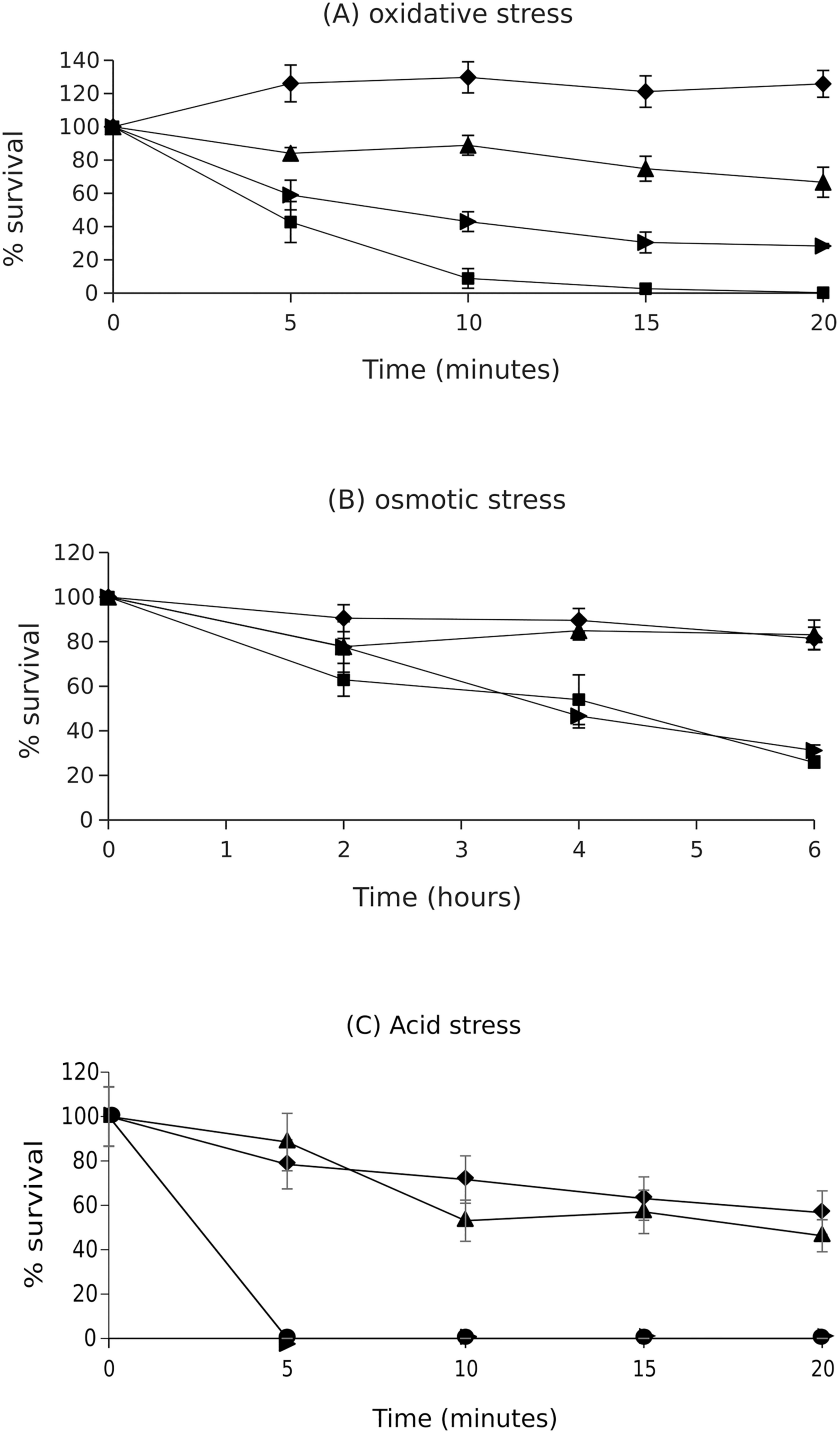
Sensitivity of E2348/69, LRT9 and their *rpoS*∷Tn*10* mutants to environmental stresses. Overnight cultures of E2348/69, LRT9 and their respective *rpoS*∷Tn*10* mutants were resuspended in (A) 0.9% NaCl supplemented with 6 mM H_2_O_2_ (oxidative stress); (B) 2 M NaCl (osmotic stress); (C) EG buffer pH 2 (acid stress). Samples were harvested at the indicated time points and plated on LB-agar for CFU counting. (♦) LRT9; (▲) E2348/69; (▶) LRT9 *rpoS*∷Tn*10*; (◼) E2348/69 *rpoS*∷Tn*10*. Each point corresponds to the mean ± SEM of three independent experiments.

To study the role of RpoS in EPEC physiology, the *rpoS* gene was knocked out in two EPEC strains—LRT9 and E2348/69. E2348/69 (O127:H6) is the EPEC prototype strain, being widely used in the study of EPEC biology and disease. LRT9 (O111:H2) is been used in our laboratory and elsewhere to study the role of regulatory genes in EPEC adherence [28, 30, 36]. Both E2348/69 and LRT9 *rpoS*∷Tn*10* mutants displayed slow bubbling in the presence of hydrogen peroxide (low catalase activity; not shown) and were also considerably more sensitive to oxidative, osmotic and acid stress (Fig 1), as expected for *rpoS*^-^negative strains.

There are two known E2348/69 strains, one of them is resistant to nalidixic acid (NalR) and the other is resistant to streptomycin (StrR). The Str resistance is due to the presence of the plasmid pE2348-2 which carries the *strAB* genes [37], while the Nal resistance was deliberately selected in the original E2348/69 StrR strain [38]. The NalR strain eventually lose plasmid pE2348-2, and consequently the resistance to streptomycin [27]. For historical reasons, the NalR strain is the one used in most laboratories. The genomes of both E2348/69 strains were sequenced and published [27, 39]. E2348/69 StrR carries a guanine insertion at position 390 of *rpoS* ORF, causing a frameshift and the emergence of premature stop codons. Polymorphisms in *rpoS* are not uncommon, some mutations are neutral while others result in a null phenotype or in an attenuated RpoS form. These include RpoS variants that are shorter or longer than the normal 38 KDa protein [7, 40–42]. The NalR strain, which is the E2348/69 variant used in this study carries a functional *rpoS* gene [27].

Given the fact that almost 50 years have passed since E2348/69 was first isolated in 1969 and since then spread in several laboratories around the world, it is not surprising to find out variations in *rpoS* in different E2348/69 stocks. In fact, nutrient limitation and prolonged growth in rich media such as LB promote the selection of *rpoS* mutants [41, 43–45]. Samples of E2348/69 StrR may have been stored in LB-stabs, a condition that encourages the emergence of *rpoS* GASP (Growth advantage in stationary phase) mutants [46]. Reacquiring of the *rpoS*^+^ allele by the NalR strain could be due to exposure to stresses such as extreme cold conditions, as happened to strain MC4100, which gained high levels of RpoS through an *rssB* mutation. Alternatively, the original StrR strain was *rpoS*^+^, while the sequenced StrR strain acquired the *rpoS* mutation later on. Compared to MG1655 (the prototype K-12 strain), the *rpoS* sequence of LRT9 revealed two amino acid substitutions: Q33E (also present in E2348/69 and very common in many K-12 and non-commensal strains [6, 7, 47, 48]) and Q306S. It is not known whether the Q306S substitution have any deleterious effect on RpoS, but this is unlikely due to the conservative nature of the substitution and also because the status of RpoS-dependent phenotypes, such as stress-resistance and strong catalase activity was quite elevated in this strain.

**Fig 2:**
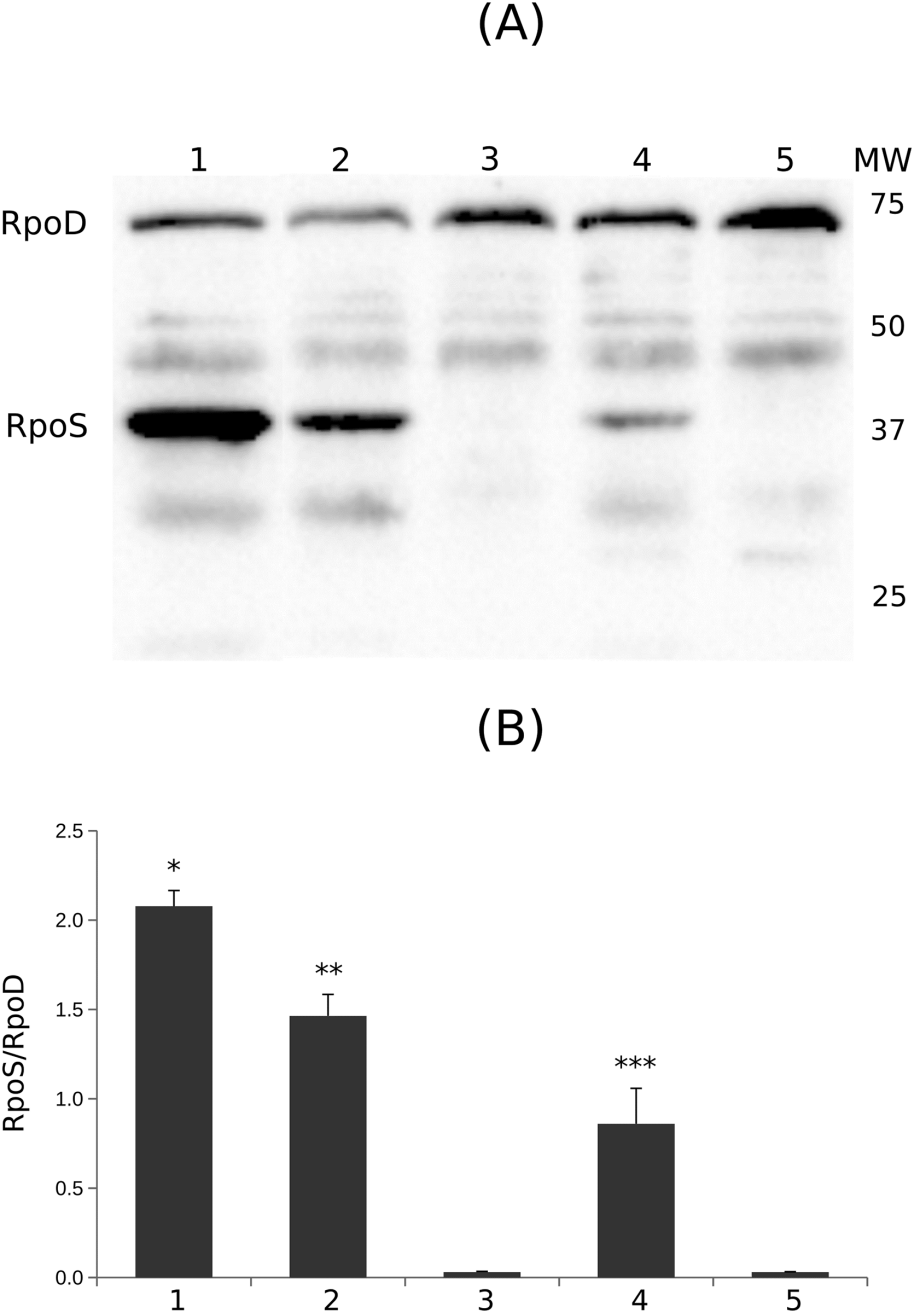
Immunoblot of RpoS in strains E2348/69 and LRT9. Total protein was extracted from bacteria grown in minimal medium supplemented with a limited concentration of Pi (0.2 mM KH_2_PO_4_) and immunoblotted against RpoS and RpoD (*σ*^70^) antibodies. (A) Representative picture of an immunoblot. (B) Quantification of RpoS normalized against RpoD for each strain. 1, MC4100; 2, LRT9; 3, LRT9 *rpoS*∷Tn*10*; 4, E2348/69; 5, E2348/69 *rpoS*∷Tn*10*. Each bar corresponds to the mean of at least three independent experiments ±SEM. Asterisks indicate statistical difference with p < 0.001 (Students’ t test).

A western-blot analysis with monoclonal RpoS antibodies confirmed that both E2348/69 and LRT9 synthesize RpoS proteins of 38 kDa (Fig 2A, as found in most *E. coli* strains, pathogenic and non-pathogenic alike. Bacteria were grown in Pi-limited medium to induce Pi-starvation, a condition which enhances the accumulation of RpoS [49]. This condition was chosen because it is similar to that used for the chemostat competition assays (see below). The level of RpoS in LRT9 was stronger than in E2348/69, but still lower than the one found in the high-RpoS strain MC4100 [9]. Similar results were obtained when bacteria were grown overnight in LB (not shown). The relatively low level of RpoS explains why E2348/69 is more sensitive to stresses than LRT9. Given that *rpoS* is regulated at several levels and by many different inputs [3, 50], we can only speculate about which of these elements contributes most to determine the level of RpoS in these strains.

### Effect of *rpoS* on adherence

Several studies have reported the contribution of RpoS to the virulence of EPEC and EHEC (for a review about the role of RpoS in pathogenesis see [19]). The role of RpoS is variable, while some of these studies claimed that RpoS plays a positive role, others have found that it reduced virulence. The main virulence trait of EPEC is its ability to bind to the small intestine epithelial cells [13, 18]. To test the effect of *rpoS* on adherence, we monitored the adherence of the wild-type strains and of their the *rpoS*∷Tn*10* mutants to HEp-2 cells, but the *rpoS* mutation did not significantly affect adherence in either LRT9 (S1A Fig) or E2348/69 (S1B Fig). Transformation of the *rpoS* mutants with pNP5 (*rpoS*^+^ low-copy plasmid) also did not have any effect on adherence. It is interesting to note that E2348/69 and its derivatives adhered more strongly than the LRT9 strains (∼3.5 × 10^7^ CFU/ml for LRT9 and ∼6 × 10^7^ CFU/ml for E2348/69).

**Fig 3:**
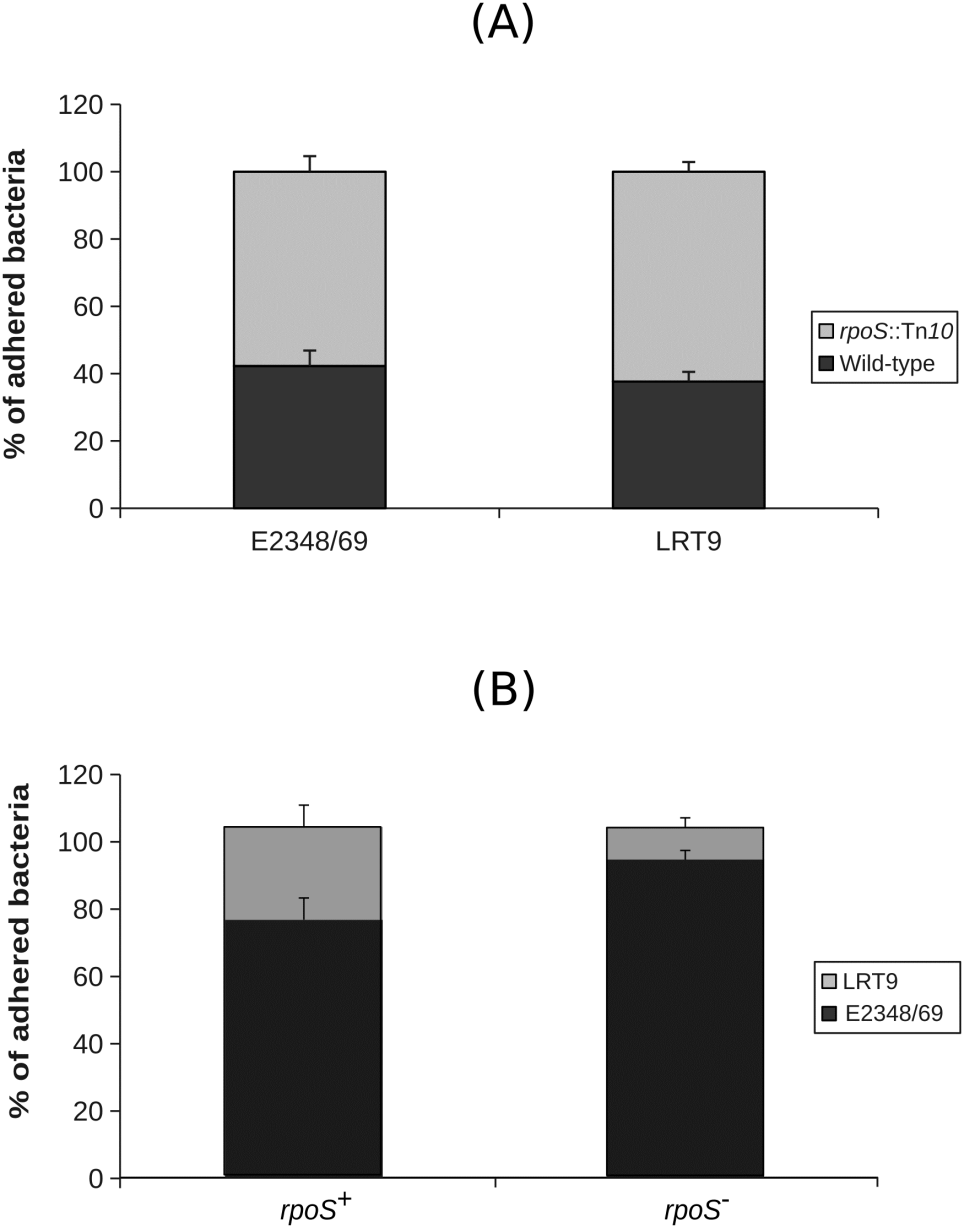
Effect of *rpoS* on the adherence of EPEC. Competition for adherence to epithelial cells between the wild-type strains (E2348/69 and LRT9) against their respective *rpoS*∷Tn*10* mutants (A) and between E2348/69 and LRT9 strains against each other (B). Bacteria were suspended in DMEM over a monolayer of HEp-2 cells for 3 hours. The adhered bacteria were then released and seeded on L-agar or on L-agar supplemented with tetracycline for CFU counting.

To further investigate this matter, competition assays between the *rpoS*∷Tn*10* mutants and their respective wild-type parents for the adherence to HEp-2 cells were conducted (Fig 3). Equal concentrations of *rpoS*^+^ bacteria (E2348/69 or LRT9) each with its respective *rpoS*∷Tn*10* mutant were suspended over a monolayer of HEp-2 cells in DMEM and incubated for three hours. The adhered bacteria were then plated on non-supplemented L-agar (non-selective medium) and on L-agar supplemented with tetracycline (selective for the *rpoS*∷Tn*10* mutant). Fig 3A shows that both *rpoS* mutants presented a slight advantage over their *rpoS*^+^ parents. 58% of the adhered E2348/69 bacteria were *rpoS*∷Tn*10* (p = 0.055), while in the case of LRT9, 63% of the adhered bacteria were *rpoS*∷Tn*10* (p = 0.0009). These results suggest that *rpoS* mutants have a small advantage in adhering to epithelial cell. The higher the RpoS intrinsic level (as in LRT9), the stronger the negative effect of the *rpoS* mutation on adherence.

When the wild-type strains competed against each other, E2348/69 adhered considerably more than LRT9 (74% E2348/69 versus 26% LRT9; p = 0.0017) (Fig 3B). To test if this advantage could be ascribed to *rpoS*, the corresponding *rpoS*∷Tn*10* mutant of each EPEC strain was set to compete against each other. The proportion of adhered E2348/69 cells increased to 91%, while only 9% of the adhered bacteria were LRT9 (p = 10^-6^). These results suggest that the advantage that E2348/69 has over LRT9 on adherence is not related to *rpoS*. To test if the advantage of E2348/69 could be ascribed to differences in growth rate, growth curves in DMEM were performed. Strain LRT9 grew better than E2348/69 in DMEM, hence the advantage of the latter over LRT9 on adherence could not be attributed to growth performance (S2 Fig).

### Effect of *rpoS* on the expression of *bfpA* and *eae*

**Fig 4:**
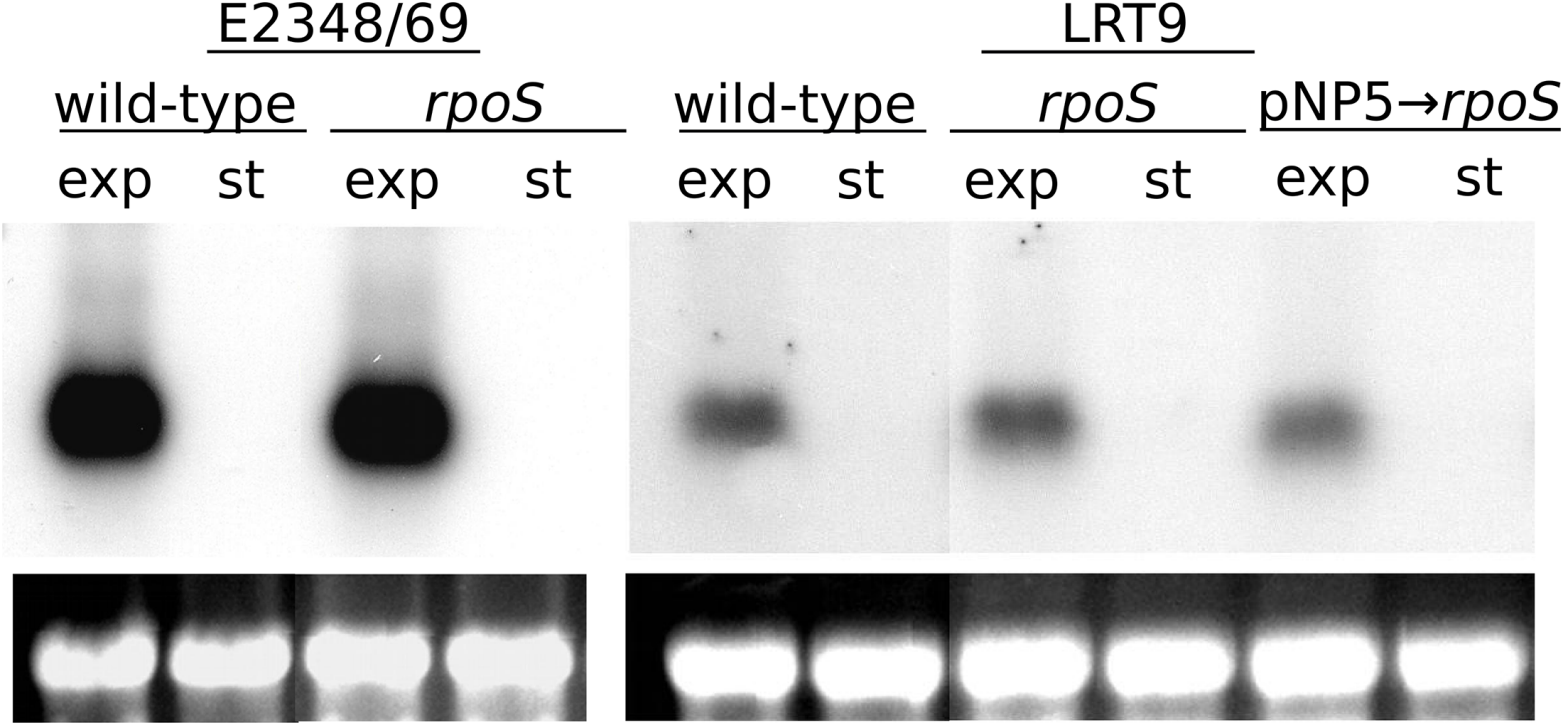
Effect of *rpoS* on the expression of *bfpA*. RNA extracted from E2348/69, LRT9, from their respective *rpoS*∷Tn*10* mutants and from the *rpoS*∷Tn*10* transformed with pNP5 (*rpoS*^+^) at the mid-exponential (exp) phase and at the beginning of the stationary phase (st) was hybridized with a labeled *bfpA* probe. The observed bands correspond to the *bfpA* transcript (0.6 Kb) and below to the 23S rRNA. The bright bands shown below the blot are the 23S rRNA stained with ethidium bromide. Each blot was repeated at least twice.

The ability of EPEC to adhere to intestinal cells depends mainly on two adhesins: type IV BFP pilus and intimin. BFP plays a fundamental role in the primary adherence of EPEC to epithelial cells [15, 51] and for that reason the effect of *rpoS* on *bfpA* transcript level was evaluated. Expression of *bfp* under the right conditions (exponentially growing cells in DME medium) is very strong and can be easily detected [14, 28]. With that aim a northern blot analysis was conducted. E2348/69, LRT9 and their *rpoS*∷Tn*10* mutants were grown in DMEM and harvested at the exponential and stationary phase. Fig 4 shows that a band corresponding to *bfpA* mRNA was observed at the exponential phase in both wild-type and *rpoS* mutants. The fact that *bfpA* mRNA was undetected at the stationary phase agrees with previous studies [14, 52]. No clear difference in the intensity of *bfpA* mRNA band could be observed when the *rpoS*∷Tn*10* mutants were compared to their *rpoS*^+^ parents, suggesting that RpoS does not affect the expression of the BFP fimbriae. Introduction of pNP5 into LRT9 *rpoS*∷Tn*10* also did not alter the transcript intensity of *bfpA*. On the other hand, the level of *bfpA* was higher in E2348/69 than in LRT9, providing a molecular basis for the observation that E2348/69 adheres more strongly to epithelial cells than LRT9 (as shown in Fig 3B and S1 Fig).

To further investigate the effect of *rpoS* on the expression of adherence-related genes, a set of experiments were conducted in strain LRT9 and in its *rpoS*∷Tn*10* mutant (S3 Fig). First, plasmids pGM30 and pGM36, which respectively carry P*bfpA*-*lacZ* and P*tir-eae*-*lacZ* (which for the sake of simplicity will be called P*eae*-*lacZ*) transcriptional fusions were transformed into LRT9 and its *rpoS*∷Tn*10* mutant. It can be observed that the *rpoS* knockout did not have any effect on the transcription of these genes. When the *rpoS* mutant carrying pGM30 was transformed with pNP5, a small increase in β-galactosidase activity was observed, but the level of β-galactosidase of the *rpoS* mutant bearing pGM36 was unchanged by the presence of pNP5. The effect of *rpoS* on the level of BfpA and intimin proteins was assessed by introducing a SPA flag at the 3’-end of *bfpA* and *eae* [30] and immunoblotting with an anti-SPA antibody. S3 Fig shows that the *rpoS* mutation did not significantly affect the expression of BfpA or intimin. Introduction of pNP5 also did not alter the level of the proteins.

### Effect of RpoS on the fitness of EPEC strains

Trade-offs are important means through which bacteria adapt to the environment and eventually promote increased bacterial diversity [53]. Allocation of resources to cell reproduction comes at the cost of neglecting the expression of proteins important for bacterial protection against environmental stresses, and vice-versa. RpoS is at the center of this trade-off: high RpoS levels promote the transcription of stress protection-related genes, while low levels of RpoS favor the transcription of vegetative (*σ*^70^-dependent) genes. To some extent the intrinsic level of RpoS determines the fitness of an *E. coli* strain [54].

**Fig 5:**
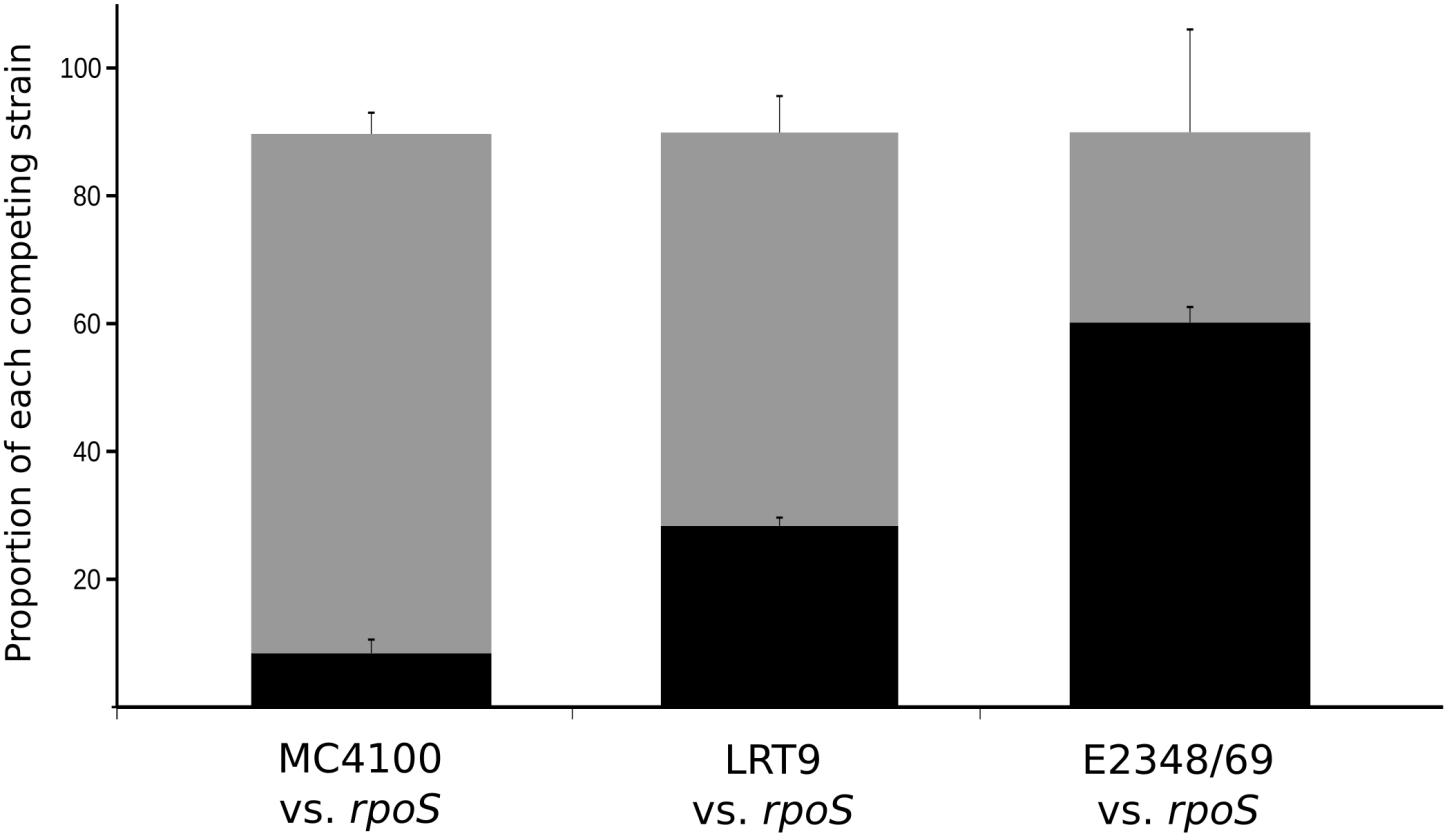
Effect of *rpoS* on EPEC fitness. MC4100, LRT9, E2348/69 were mixed at 1:1 ration with their respective *rpoS*∷Tn*10* mutants and grown for 24 hours in a chemostat with minimal medium containing 0.2% glucose and 30 μM KH_2_PO_4_ at a dilution rate of 0.1 h^-1^. Samples were removed, diluted and plated on L-agar supplemented or not with tetracycline for CFU determination. Black color, wild-type strains; grey color, *rpoS*∷Tn*10* mutants. Bars represent the mean ±SEM of three independent competitions.

To evaluate the contribution of RpoS to the fitness of E2348/69 and LRT9 competitions between each wild-type strain and its respective *rpoS* mutant were set up in a chemostat under continuous growth. As a control, strain MC4100 that has a high endogenous RpoS level was set to compete against its *rpoS* mutant. Equal numbers of bacteria were inoculated in a chemostat containing minimal medium (TGP) supplemented with a limited concentration of phosphate (30 μM) and grown for 24 h. Fig 5 shows that while MC4100 *rpoS*∷Tn*10* outcompeted the wild-type strain by a 10:1 factor, the effect of the *rpoS*∷Tn*10* allele in LRT9 was less potent, but still the *rpoS*∷Tn*10* mutant performed better than the wild-type strain. Conversely, the *rpoS* mutant of E2348/69 was slightly outcompeted by the wild-type strain, but the difference was not statistically significant (p = 0.13). Even so, it is quite surprising that the *rpoS* mutation did not confer any advantage under nutrient-limited conditions in this strain. Hence, unlike the MC4100 and LRT9 strains, *rpoS* did not have a clear deleterious effect on the fitness of E2348/69 under nutritional limitation. The reason for it may reside in the fact that E2348/69 bear low levels of endogenous RpoS, which in turn is not sufficient to tilt the SPANC balance towards the transcription of RpoS-dependent genes [45]. Accordingly, strain E2348/69, which displayed the lowest level of RpoS among the three strains tested here is the most sensitive to environmental stresses, but is the least affected by RpoS regarding nutritional stress, exactly as predicted by the trade-off hypothesis [55].

## Discussion

The SPANC balance predicts that a bacterium cannot simultaneously be nutritionally competent and highly resistant to stresses [5]. High levels of RpoS may confer on the bacterium strong resistance against stresses, but can also reduce the expression of *σ*^70^-dependent genes [14, 16]. However, the level of RpoS in E2348/69 and LRT9 was not as high as to compromise its ability to adhere to the cell host. Once *rpoS* is essential for a proper acid response and does not significantly affect adherence, it is advantageous for the bacterium to keep a functional *rpoS* gene. In fact, most natural isolates of *E. coli*, pathogenic and non-pathogenic alike, carry wild-type alleles of *rpoS* [56–58]. However, *rpoS* normally have a negative effect on fitness, as shown for strain MC4100 and to a lesser extent for LRT9. On the other hand, the presence of *rpoS* in strain E2348/69 was not disadvantageous, meaning that by keeping *rpoS* the bacterium did not have to trade stress resistance for nutritional competence, as stipulated by the SPANC balance [53].

Competition between *rpoS*^+^ and *rpoS*^-^ strains in the intestine should be common, because different *E. coli* strains, as well as other species, with different genetic backgrounds may be present simultaneously competing with each other either for nutritional resources or for adherence to different substrates [59]. Given the readiness in which *E. coli* under continuous growth acquire mutations in *rpoS* [45, 49], it is conceivable that *rpoS* mutants might be selected in the intestine, which operates under analogous conditions [60], i.e., steady-state growth under limited concentration of one or more nutrients.

There are several reports with conflicting results about the effect of *rpoS* on EHEC and EPEC virulence. RpoS was shown to have a positive effect on the adherence of the EHEC strain EDL933 [6] and on the expression of LEE3 (LEE3-*lacZ* fusion in a K-12 background) [20] and LEE4 (*esp* operon fusion to *lacZ* in a K-12 background) [21]. Conversely, the *rpoS* mutation in EHEC Sakai strain enhanced the transcription of the LEE operons [22] and over-expression of *rpoS* in EDL933 strongly repressed the expression of LEE4 [23]. The protein levels of Tir and EspA were enhanced by a *rpoS* mutation in EDL933 [6]. In *Citrobacter rodentium*, the level of transcription of all LEE operons was enhanced by *rpoS* [6]. Finally, Hansen et al. [24] have shown that overexpression of *rpoS* in an EHEC *hfq* mutant did not affect the expression of *espB* and *tir*. In summary, there is no consensus about the role of *rpoS* in LEE expression and on adherence to epithelial cells. Altogether, the data presented here suggest that RpoS does not interfere with EPEC adherence, but it plays a positive role in bacterial protection against stresses, enhancing survival in a hostile environment, such as the mammalian stomach.

Overall the E2348/69 strain behaves exactly as if the SPANC balance was shifted to lower resistance/more fitness. E2348/69 produces less RpoS than LRT9 and is more sensitive to environmental stresses. On the other hand, E2348/69 displayed an enhanced ability to adhere to epithelial cells, at least in part due to a stronger expression of the *bfp* operon, which is controlled by a *σ*^70^-dependent promoter. However, the differential level of adherence and BFP expression in E2348 and LRT9 cannot be ascribed to *rpoS*, because the competitive advantage of E2348/69 *rpoS*∷Tn*10* over LRT9 *rpoS*∷Tn*10* was even stronger than between the *rpoS*^+^ parents. The SPANC balance is often related to cellular levels of RpoS, as high-levels of RpoS confer high resistance to stresses but are antagonized by a poor ability to utilize alternative nutrient sources [5]. The trade-off that explains the dichotomy between growth and survival also applies here in the case of strain LRT9, where the confrontation is between nutritional competence (bacterial fitness) and stress resistance. Interestingly, the ability to colonize the host (adherence to epithelial cells), which is also regulated by *σ*^70^-dependent genes was not subject to a RpoS-related trade-off. The strong advantage of E2348/69 over LRT9 in expressing adhesins and adhering to epithelial cells must be due to other genetic components.

Introduction of the *rpoS*∷Tn*10* mutation in both EPEC strains was carried out by P1 transduction from a K-12 strain. The DNA region downstream of *rpoS* in many EPEC isolates differs from that of K-12 strains by the presence of a 2.9 Kb sequence harboring three ORFs, *hosA*, *pad1* and *yclC* [61]. *hosA* encodes a transcriptional regulator that belongs to the SlyA family; *pad1* codes for a phenylacrylic acid decarboxylase, that confers resistance to phenylacrylic acids and *yclC* encodes the C subunit of a phenolic acid decarboxylase. Both E2348/69 and LRT9 carry the 2.9 Kb additional stretch of DNA. Upon transduction of the *rpoS*∷Tn*10* mutation the *rpoS* + 2.9 Kb segment was replaced by the K-12 *rpoS* region (data not shown). To certify that the effect or lack of effect of the *rpoS*∷Tn*10* mutation was due to *rpoS* and not to one of the genes contained in the 2.9 Kb region, the *rpoS*∷Tn*10* mutants were transformed with the low-copy plasmid pNP5 which bears a wild-type copy of *rpoS* [32]. In all relevant phenotypes, such as adherence to HEp-2 cells, *bfpA* and *eae* transcription, and BfpA and Intimin protein levels, no effect of *rpoS*∷Tn*10* was recorded. Complementation with pNP5 also did not significantly alter the behavior of EPEC regarding those phenotypes. These results also indicate that the 2.9 Kb region downstream of *rpoS* does not exert any influence on EPEC adherence. These findings are in agreement with [62] that showed that *hosA*, which is located immediately downstream of *rpoS* in EPEC and EHEC, did not affect type III secretion, LEE1 and LEE4 regulation, or the ability of E2348/69 to form attaching-and-effacing lesions on intestinal epithelial cells.

## Conclusion

RpoS plays a mostly positive role in EPEC biology. The positive effect of *rpoS* on bacterial resistance against environmental stresses in both E2348/69 and LRT9 strains was unambiguous. The cost of carrying and expressing *rpoS* was non-existent in the case of E2348/69 and modest in the case of LRT9. RpoS levels were higher in strain LRT9 than in strain E2348/69, which was consistent with the stronger protection against oxidative stress observed in strain LRT9 and the negative effect that *rpoS* had on the fitness of this strain. The ability to adhere to epithelial cells, which is the most relevant EPEC trait, was not significantly affected by RpoS in either strain.

## Supporting information

S1 Fig Effect of *rpoS* on the adherence of EPEC to epithelial cells. 5 × 10^7^ bacteria were transferred to HEp-2 cells monolayers in DMEM supplemented with 2% FBS and incubated for 3 h. The cell wells were washed and the bacteria were released, diluted and plated on L-agar for CFU counting. (A) LRT9, wild-type strain; *rpoS*∷Tn*10*, LRT9 carrying a *rpoS* mutation; pNP5->*rpoS*∷Tn10, p*rpoS*^+^ plasmid in strain LRT9 *rpoS*∷Tn*10*. (B) E2348/69, wild-type strain; E2348/69 *rpoS*∷Tn*10*; pNP5-¿E2348/69 *rpoS*∷Tn10. Each bar represents the mean ± S.E.M. of three independent experiments.
(PDF)

S2 Fig Growth curves of strains E2348/69, LRT9 and their *rpoS* mutants. Bacteria grown overnight were diluted in DMEM and grown for 9 hours. Samples were taken hourly and monitored for cell density at OD_600_. The growth rates for exponentially growing E2348/69, E2348/69 *rpoS*∷Tn*10*, LRT9 and LRT9 *rpoS*∷Tn*10* strains were, respectively, 0.48 h^-1^, 0.99 h^-1^, 0.66 h^-1^ and 1.0 h^-1^. Each point represents the mean of three independent cultures.
(PDF)

S3 Fig Effect of *rpoS* on the expression of *bfpA* and *eae* in strain LRT9. Bacteria were grown in DME medium and incubated at 37℃ without shaking for 6 hours, at which time samples were withdrawn and assayed for (A) β-galactosidase. pGM30, operon fusion between *bfpA* promoter and *lacZ*; pGM36, *tir-eae* promoter fused to *lacZ*. WT, strain LRT9; *rpoS*, LRT9 *rpoS*∷Tn*10* and pNP5→*rpoS*, LRT9 *rpoS*∷Tn*10* carrying plasmid pNP5. (B) Bacteria grown as described above were harvested and immunoblotted with anti-SPA (FLAG 3X) antibodies. WT, *rpoS* and pNP5 *rpoS* correspond to LRT9 and its derivatives carrying chromosomal copies of *bfpA*∷SPA or *eae*∷SPA.
(PDF)
