# Supplementary material for: RpoS role in virulence and fitness in enteropathogenic Escherichia coli
Source: PLoS One. 2017 Jun 29;12(6):e0180381. doi: 10.1371/journal.pone.0180381 (PMC5491219; doi:10.1371/journal.pone.0180381)
Supplement: S1 Fig — 5 × 10^7^ bacteria were transferred to HEp-2 cells monolayers in DMEM supplemented with 2% FBS and incubated for 3 h. The cell wells were washed and the bacteria were released, diluted and plated on L-agar for CFU counting. (A) LRT9, wild-type strain; rpoS∷Tn10, LRT9 carrying a rpoS mutation; pNP5->rpoS∷Tn10, prpoS+ plasmid in strain LRT9 rpoS∷Tn10. (B) E2348/69, wild-type strain; E2348/69 rpoS∷Tn10; pNP5-¿E2348/69 rpoS∷Tn10. Each bar represents the mean ± S.E.M. of three independent experiments. (PDF) [file pone.0180381.s001.pdf]

(A)

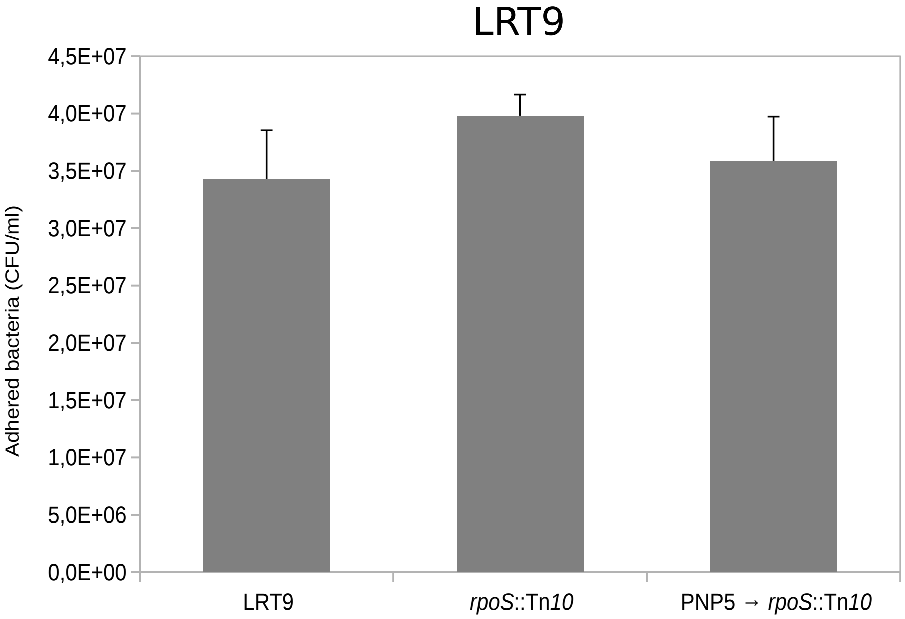

(B)

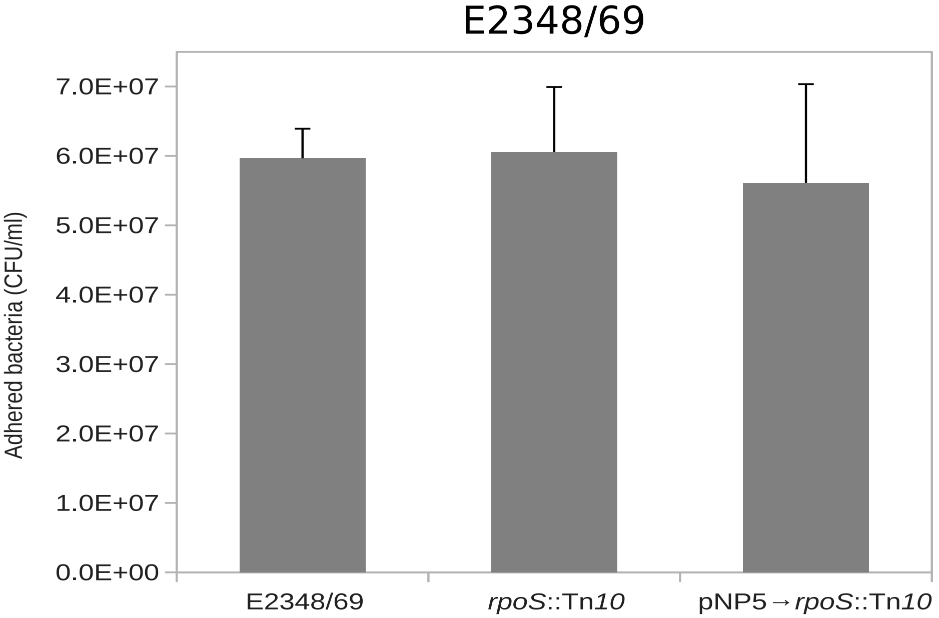

Figure S1 Effect of *rpoS* on the adherence of EPEC to epithelial cells.

5×10^7 bacteria were transferred to HEp-2 cells monolayers in DMEM supplemented with 2% FBS and incubated for 3 h. The cell wells were washed and the bacteria were released, diluted and plated on L-agar for CFU counting. (A) LRT9, wild-type strain; *rpoS*::Tn*10*, LRT9 carrying a *rpoS* mutation; pNP5->*rpoS*::Tn10, p*rpoS*$^+$ plasmid in strain LRT9 *rpoS*::Tn*10*. (B) E2348/69, wild-type strain; E2348/69 *rpoS*::Tn*10*; pNP5->E2348/69 *rpoS*::Tn10. Each bar represents the mean ± S.E.M. of three independent experiments.
