# Supplementary material for: RpoS role in virulence and fitness in enteropathogenic Escherichia coli
Source: PLoS One. 2017 Jun 29;12(6):e0180381. doi: 10.1371/journal.pone.0180381 (PMC5491219; doi:10.1371/journal.pone.0180381)
Supplement: S2 Fig — Bacteria grown overnight were diluted in DMEM and grown for 9 hours. Samples were taken hourly and monitored for cell density at OD600. The growth rates for exponentially growing E2348/69, E2348/69 rpoS∷Tn10, LRT9 and LRT9 rpoS∷Tn10 strains were, respectively, 0.48 h-1, 0.99 h-1, 0.66 h-1 and 1.0 h-1. Each point represents the mean of three independent cultures. (PDF) [file pone.0180381.s002.pdf]

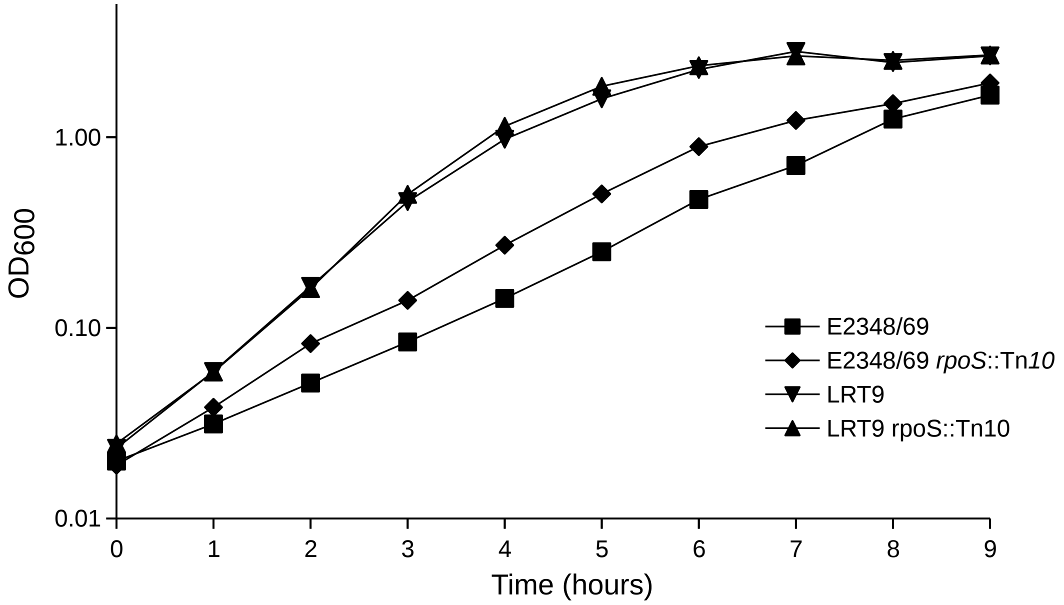

Figure S2 Growth curves of strains E234869, LRT9 and their *rpoS* mutants. Bacteria grown overnight were diluted in DMEM and grown for 9 hours. Samples were taken hourly and monitored for cell density at OD_{600}. The growth rates for each strain in the exponential phase were 0.48 h^{-1}, 0.99 h^{-1}, 0.66 h^{-1} and 1.0 h^{-1} for E2348/69, E2348/69 *rpoS*::Tn*10*, LRT9 and LRT9 *rpoS*::Tn*10*, respectively. Each point represents the mean of three independent cultures.
