# Supplementary material for: RpoS role in virulence and fitness in enteropathogenic Escherichia coli
Source: PLoS One. 2017 Jun 29;12(6):e0180381. doi: 10.1371/journal.pone.0180381 (PMC5491219; doi:10.1371/journal.pone.0180381)
Supplement: S3 Fig — Bacteria were grown in DME medium and incubated at 37℃ without shaking for 6 hours, at which time samples were withdrawn and assayed for (A) β-galactosidase. pGM30, operon fusion between bfpA promoter and lacZ; pGM36, tir-eae promoter fused to lacZ. WT, strain LRT9; rpoS, LRT9 rpoS∷Tn10 and pNP5→rpoS, LRT9 rpoS∷Tn10 carrying plasmid pNP5. (B) Bacteria grown as described above were harvested and immunoblotted with anti-SPA (FLAG 3X) antibodies. WT, rpoS and pNP5 rpoS correspond to LRT9 and its derivatives carrying chromosomal copies of bfpA∷SPA or eae∷SPA. (PDF) [file pone.0180381.s003.pdf]

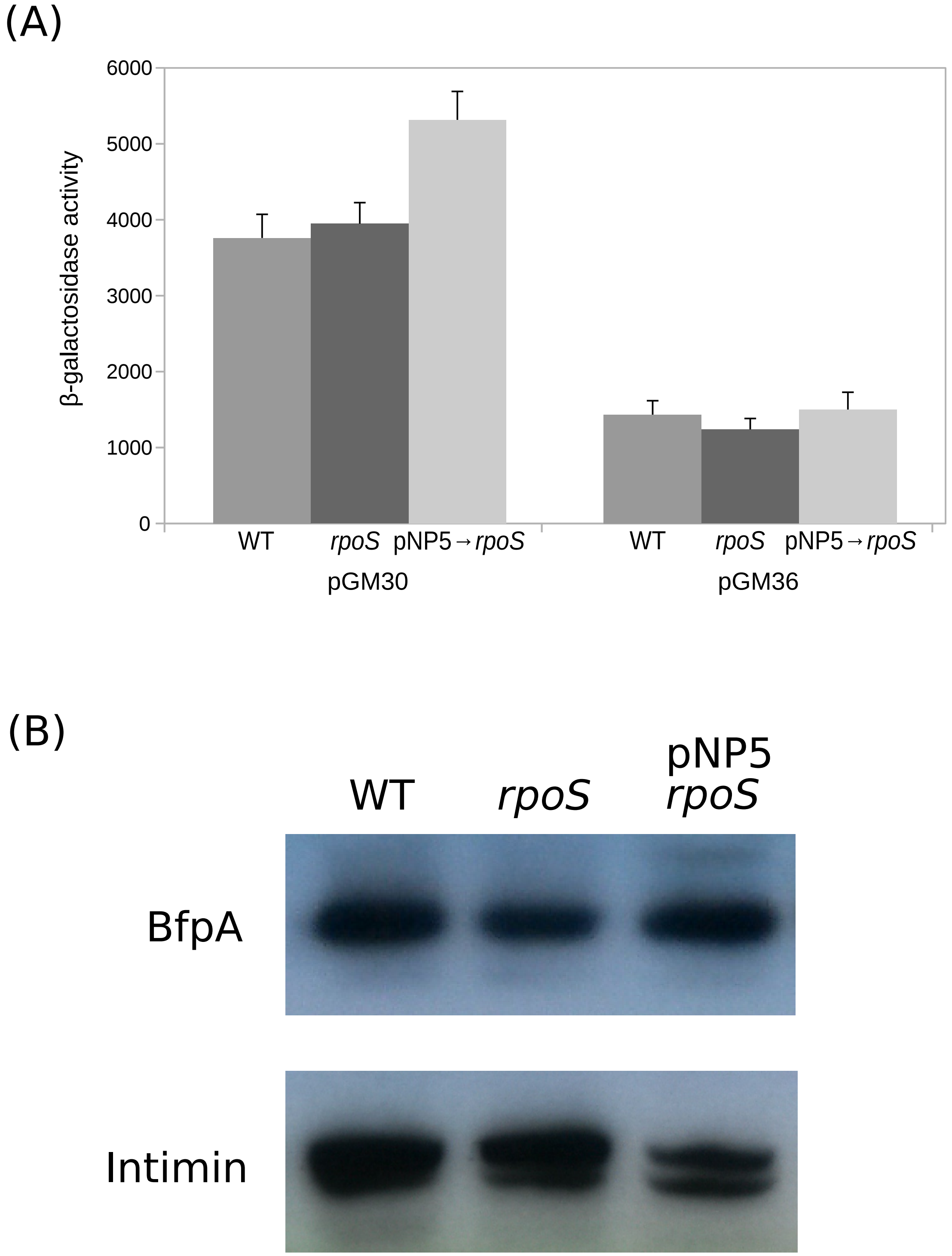

Figure S3 Effect of *rpoS* on the expression of *bfpA* and *eae* in strain LRT9. Bacteria were grown in DME medium and incubated at 37°C without shaking for 6 hours, at which time samples were withdrawn and assayed for (A) β-galactosidase. pGM30, operon fusion between *bfpA* promoter and *lacZ*; pGM36, *tir-eae* promoter fused to *lacZ*. WT, strain LRT9; *rpoS*, LRT9 *rpoS*::Tn*10* and pNP5→*rpoS*, LRT9 *rpoS*::Tn*10* carrying plasmid pNP5. (B) Bacteria grown as described above were harvested and immunoblotted with anti-SPA (FLAG 3X) antibodies. WT, *rpoS* and pNP5 *rpoS* correspond to LRT9 and its derivatives carrying chromosomal copies of *bfpA*::SPA or *eae*::SPA.
